# Urban parks as social infrastructure: behavioral pathways to social interaction in densifying cities

**DOI:** 10.3389/fpubh.2026.1892114

**Published:** 2026-09-16

**Authors:** Noman Sahito, Omar Almahdy, Muhammad Aamir Basheer, Amjad Ali, Zulfiqar Ali Lashari, Farrukh Baig

**Affiliations:** 1 Department of Architecture and City Design, College of Design and Built Environment, King Fahd University of Petroleum and Minerals, Dhahran, Saudi Arabia; 2 Interdisciplinary Research Center for Sustainable Energy Systems (IRC-SES), King Fahd University of Petroleum and Minerals, Dhahran, Saudi Arabia; 3 Interdisciplinary Research Center for Smart Mobility and Logistics, King Fahd University of Petroleum and Minerals, Dhahran, Saudi Arabia; 4 Department of Electrical Engineering, King Fahd University of Petroleum & Minerals, Dhahran, Saudi Arabia; 5 Department of City and Regional Planning, Mehran University of Engineering and Technology, Jamshoro, Pakistan

**Keywords:** healthy aging, older adults, public space, social interaction, theory of planned behavior, urban parks, Inclusive Planning

## Abstract

**Background:**

Urban parks are important public health and social infrastructure, yet evidence on how older adults’ park-use intentions are associated with visitation and Social interaction remains limited, particularly in densely populated Global South cities. This study applied an extended Theory of Planned Behavior (TPB) framework to examine behavioral and contextual factors associated with park use and social interaction among older adults in Karachi, Pakistan.

**Methods:**

A questionnaire survey was conducted among 576 residents aged 50 years and older. Partial least squares structural equation modeling (PLS-SEM) was used to examine behavioral intentions, self-reported park-use behavior, social interaction in parks (SIP), and contextual antecedents of perceived behavioral control (PBC).

**Results:**

Attitude (*β* = 0.543, *p* < 0.001), subjective norms (*β* = 0.516, *p* < 0.001), and PBC (*β* = 0.306, *p* < 0.001) were positively associated with behavioral intention. Behavioral intention was positively associated with park-use behavior (*β* = 0.369, *p* < 0.001). Park-use behavior (*β* = 0.469, *p* < 0.001) and behavioral intentions (*β* = 0.372, *p* < 0.001) were positively associated with SIP. Government/community support (*β* = 0.445, *p* < 0.001) and facilities/accessibility (*β* = 0.308, *p* < 0.001) were positively associated with PBC. Park-use behavior significantly mediated the association between behavioral intention and SIP.

**Conclusion:**

Older adults’ park use and social interaction were associated with attitudes, social norms, perceived control, and supportive physical and governance conditions. The findings suggest that urban parks can function as age-supportive social infrastructure when accessible facilities are complemented by reliable maintenance, safety measures, community support, and initiatives encouraging regular park visitation and social engagement.

## 1 Introduction

The urban environment, as well as population aging, is one of the defining processes that characterize city development in the twenty-first century (1). The concept of age-friendly city policy recognizes the need for cities to enable older persons to be active, mobile, and socially engaged by overcoming barriers and providing environments that facilitate this (1). Demographic projections indicate that the global population aged 65 years and above will increase substantially by mid-century, intensifying the need for urban environments that support older adults’ mobility, wellbeing, participation, and social inclusion (2). Consistent with SDG Target 11.7, global urban policy calls for universal access to safe, inclusive, and accessible green and public spaces, particularly for older persons, women, children, and persons with disabilities (3, 4).

One prominent public infrastructure that could help promote healthy aging in urban settings is urban parks in densely populated cities (5). Urban parks can offer low-cost recreational spaces that are close and provide opportunities to engage in physical activities (6). Accumulating empirical evidence demonstrates that both park infrastructure and its quality (in terms of accessibility and amenities) can influence wellbeing outcomes and experiences among older persons. Parks, however, are more than just a health infrastructure; they are also a social infrastructure (7, 8). Urban parks can be viewed as spaces of encounter, as places where social encounters can be facilitated through co-presence and repeated visits (9), Reviews on public space and social cohesion reveal that physical attributes (such as presence of seating, furniture and mixed uses), and perceptions of places (for example, feelings of safety and comfort) shape the extent to which public spaces promote social interaction and social cohesion, as well as the social patterning of the effects, notably by socio-demographic characteristics, such as age (9, 10).

Nevertheless, an essential challenge remains: the health- and socially beneficial impact of parks depends on older individuals’ park visitation. Despite parks being available, older adults may face mobility constraints, safety concerns, access restrictions, inadequate facilities, and a perceived lack of control over park use (11, 12). This issue is especially important in dense cities of the Global South, where green space is often contested, unevenly maintained, and embedded in wider constraints relating to crowding, mobility, insecurity, infrastructure deficits, and variable governance. The Karachi city was selected as a case study, Pakistan’s largest metropolitan city, with a population of exceeding 20.3 million (13). Studying older adults’ engagement with parks in this setting is valuable because it extends the evidence beyond the heavily studied East Asian, European, and North American contexts and tests whether age-friendly park-use pathways operate similarly in a dense South Asian metropolis. Hence, the study of park visitation behavior in densely populated cities requires theoretically grounded models that elucidate how older adults intend to visit parks, turn those intentions into practice, and gain opportunities for social interaction as a result (11, 14).

The Theory of Planned Behavior (TPB) appears to be quite promising for addressing this task. TPB presupposes that any particular behavior occurs subsequent to the formation of a behavioral intention, determined by attitude towards the behavior, subjective norms, and perceived behavioral control (15, 16). TPB has already been utilized in park/urban green space-related research to predict demand for park services and park use patterns, including extended versions considering park accessibility and constraints to park use (17). Nevertheless, this body of literature mainly focuses on either intention to visit parks or park use behavior, rather than on subsequent social interactions (17). On the contrary, another literature stream studying the social aspects of parks explores the role of use and use patterns in shaping the relationship between social cohesion and parks. Thus, there is a need to integrate TPB models of park intention-behavior relationships with models of social interaction (9).

This study addresses this problem through an extended TPB framework. In the Theory of Planned Behavior, intention is shaped by attitude, subjective norms, and perceived behavioral control, and intention is the most proximal predictor of behavior (15, 16). That framework is useful here because urban park visitation among older adults is partly motivational and partly conditional: residents may value parks, but actual use also depends on whether they feel able to access and use them safely and comfortably (11, 17). Prior park studies have shown that TPB can help explain older adults’ intentions to use parks and their self-reported park use. However, the literature remains less integrated regarding downstream social outcomes (11, 14). Work on public space and green space often examines social cohesion or social interaction separately from TPB-based intention–behavior models, even though the two literatures are logically connected (9, 18, 19).

This study, therefore, advances a more integrated behavioral-public-health framework. It examines whether attitude toward visiting urban parks, subjective norms, and perceived behavioral control are associated with behavioral intention; whether intention is associated with park-use behavior; whether behavioral intention and park-use behavior are associated with social interaction in parks; and whether park-use behavior statistically mediates the intention–interaction association. In addition, it extends TPB by positioning facilities/accessibility and government/community support as contextual antecedents of perceived behavioral control, thereby making the model more responsive to the realities of park use in dense urban settings. The paper’s contribution lies not in claiming causal effects from cross-sectional data, but in offering a theoretically structured account of how age-friendly park use may relate to social inclusion in an understudied Global South context with direct relevance for public health and urban policy (20, 21).

## 2 Theory of planned behavior and theoretical framework

TPB is an extension of the Theory of Reasoned Action (TRA) and has been widely validated in predicting behavioral intentions and behaviors among humans (15, 16). In this study, the TPB model will be employed to describe the behavioral process for urban park use by older adults in Karachi. Urban park usage is a repetitive daily activity that requires certain level of motivation (such as the favorable evaluation of visiting urban parks) and feasibility (mobility, time, perception of availability, safety and usability of urban parks) (14, 17). Such criteria match well with TPB framework as old residents’ intention to visit urban parks is expected to be high in individuals who (i) believe that visiting parks will benefit and please them (attitude), (ii) perceive that important people in their life would support or encourage them to visit parks (subjective norm), and (iii) believe that visiting parks was feasible given their personal situations and constraints (perceived behavioral control) (14, 15).

One key construct in TPB is Perceived Behavioral Control (PBC), since urban park visits in crowded cities are rarely entirely voluntary (14, 15). As mentioned earlier, Ajzen later elaborated that PBC has self-efficacy and controllability components, and that the measurement should include both (22). Park visits of the older generation can be affected by enabling or restraining environmental factors, such as the availability of seating and rest areas in parks, safe pedestrian routes to reach the park, park maintenance, and perceptions of the safety and organization of parks (8, 12).

Accordingly, this study specifies facilities and accessibility (FAC) and government and community support (GOV) as contextual antecedents of PBC (14, 17). FAC captures perceived age-relevant usability conditions (e.g., adequate seating and rest areas, ease of access, age-friendly facilities), which are repeatedly highlighted in park research as shaping the accessibility and experience of park visitation (23, 24). GOV captures perceptions of whether local authorities and community actors maintain parks, support usability, and contribute to safety and upkeep conditions that can reduce barriers and raise perceived control over visiting (23, 25). This specification preserves TPB’s core structure (ATT/SN/PBC → intention→behavior) while making the control component more context-sensitive to dense-city realities (15, 22).

Beyond predicting park-use behavior, the present framework, as shown in Figure 1, extends the pathway to a downstream social outcome: social interaction in parks (SIP). In age-friendly urban perspectives, maintaining social connections and everyday participation are central to healthy aging, and public spaces are often the settings where casual encounters and repeated co-presence occur (1, 11, 26). Parks are empirically studied for their potential to promote social interaction among older adults and for how park characteristics affect the nature of that interaction. Thus, SIP is theorized as associated with both (a) park behavior in terms of actual park utilization (since there must be some park behavior in order to engage in interaction) and (b) behavioral intention (since intention could be a planned effort for utilizing the park and, in particular, meeting other individuals) (11, 14, 15).

**Figure 1:**
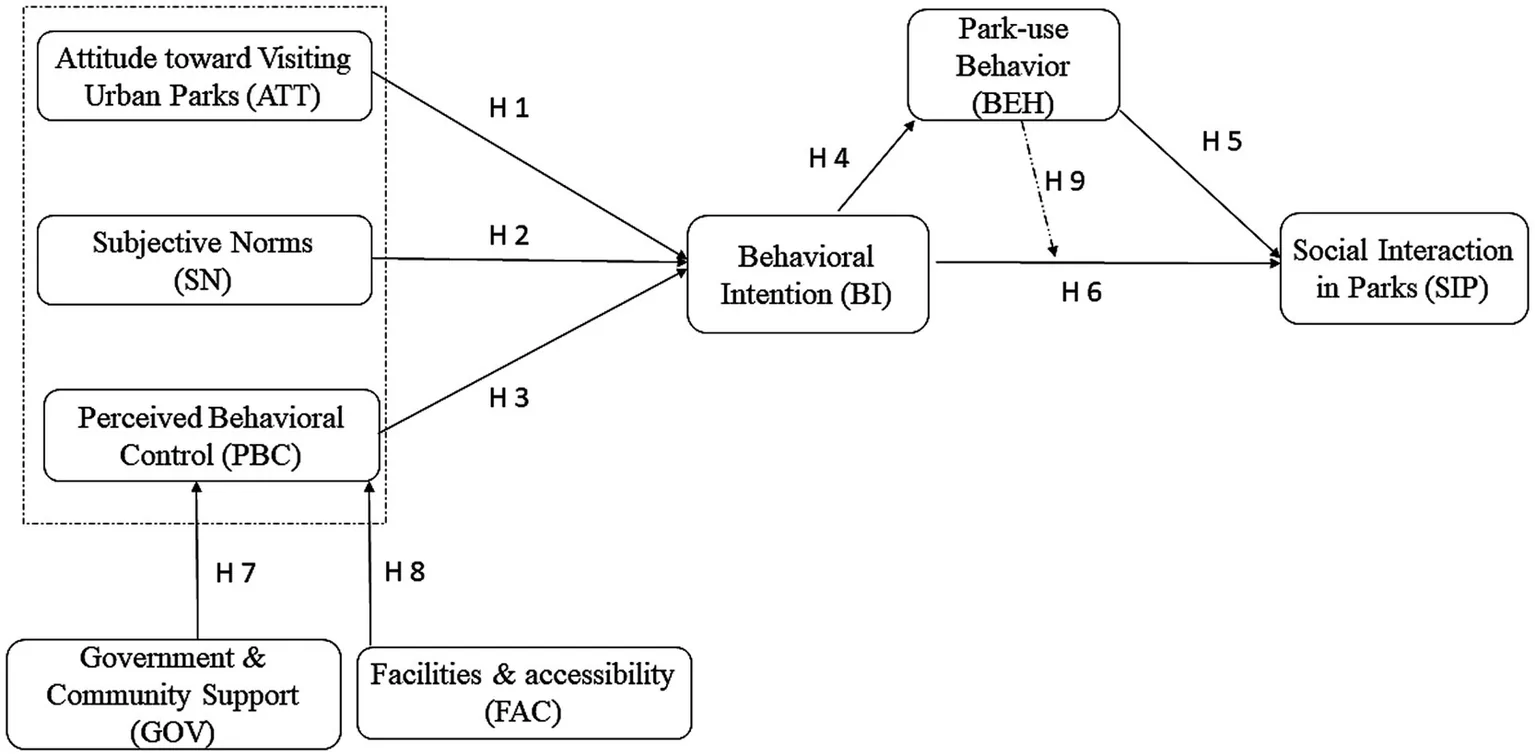
Proposed research framework.

Accordingly, the proposed model specifies that BI is associated with BEH, which, in turn, is associated with SIP (11, 14, 15). In other words, the mediation process aligns with findings on the influence of use patterns on social interaction in public spaces (9, 14, 18). At the same time, retaining a direct BI→SIP path is reasonable because intention can capture motivational orientations toward social engagement and planned visitation that may influence interaction propensity beyond frequency/time (e.g., intentionally visiting parks to meet others rather than visiting only for solitary restoration) (15, 16). This structure also reflects broader public-space evidence that social cohesion and interaction emerge through an interplay of motivations, perceptions, and use, rather than through use alone (9, 19).

### 2.1 Attitude toward visiting Urban Park

In TPB, attitude toward the behavior refers to an individual’s overall evaluation of performing a specific behavior in this case, visiting urban parks (15). Attitude reflects the extent to which visiting parks is perceived as enjoyable, beneficial, and worthwhile (15, 27). Positive attitudes generally increase behavioral intention because individuals prefer actions that are expected to yield valued outcomes (15, 16). TPB conceptualizes this link as one of the central determinants of intention, and meta-analytic evidence supports attitude as a consistent predictor of intention across many behavioral domains (15, 16). In park research, attitudes toward parks and the perceived value of park visitation have been incorporated into expanded TPB models predicting park-use demand and behavior; such work suggests that positive perceptions and liking of parks can strengthen intention and, through intention, influence park-use outcomes (14, 17).

For older adults, park-related attitudes are likely shaped by perceived well-being benefits and the expected pleasantness of the park environment (7, 8). Evidence on older adults indicates that park quality, accessibility, and environmental perceptions relate to well-being outcomes and experiences, implying that parks can be evaluated as resources for everyday functioning and mood (7, 24). In dense urban contexts, where heat, congestion, and limited private open space can constrain everyday outdoor leisure, the perceived value of parks as “breathing spaces” may increase the salience of attitude in intention formation. At the same time, negative beliefs (e.g., crowding or discomfort) could reduce positive attitudes, lowering intention (14, 15).

*H1:* Attitude toward visiting urban parks (ATT) is positively associated with behavioral intention (BI).

### 2.2 Subjective norms

Subjective norms represent social pressure, approval, or encouragement from relevant others (relatives, friends, neighbors, peers) regarding whether an individual should engage in a particular behavior (15, 16). According to TPB, people are expected to have stronger behavioral intentions if they feel that relevant others approve of their performing the behavior (15). Normative influence can play a role, especially among older adults who visit parks as part of their family duties or relationships, for caregiving tasks (such as accompanying grandchildren), or for socialization with peers (5, 28). Findings from the literature on older adults’ engagement with green space indicate that social networks and social embedding can be essential factors affecting the frequency of park visits (5). For instance, research on urban green spaces as spaces of encounter indicates that social integration into networks is associated with older adults’ park visits, suggesting that social relations can influence park use among seniors (5, 29).

In the context of Karachi, subjective norms could include cultural norms regarding family leisure activities, neighborhood walks, as well as mobility and safety of older adults. When older adults may hesitate to go outside by themselves, social approval and encouragement can serve as social enablement, leading to behavioral intentions regardless of park access availability (14, 15).

*H2:* Subjective norms (SN) are positively associated with behavioral intention (BI).

### 2.3 Perceived behavioral control (PBC)

Perceived Behavioral Control refers to perceived capability and ease/difficulty of engaging in the behavior, including beliefs about both resources and obstacles. This construct covers both perceived self-efficacy and perceived controllability. According to TPB, higher perceived control leads to greater intentions to engage in the behavior since people want to do whatever they perceive themselves as being able to do (15, 22). Several meta-analyses provide evidence that PBC is a strong predictor of intention and, in many cases, behavior (16). In regard to parks and cities research, empirical evidence indicates that perceived accessibility and constraint-related variables play a role in park-use demands, intentions, and behaviors within extended versions of TPB, underscoring the importance of control beliefs related to feasibility and accessibility for visiting a park (17).

As for older adults, their perceptions of behavioral control over visiting a park depend on such factors as available time, mobility, transportation means, as well as perceived usability of the park environment (i.e., presence of seating, shade, restrooms, paved paths, etc.) (30, 31). Research shows that park accessibility and its attributes can affect relationships among environmental perceptions, leisure activity frequency, and psychological well-being among older adults; thus, the feasibility of park visits is essential (32). Furthermore, visitor studies show that proximity, trail/park condition, maintenance, and security are the key access-related issues, suggesting that control beliefs are shaped by the environment in addition to the motivation to visit a park (12, 23).

Thus, in Karachi, where parks vary in quality and maintenance, and older adults may face difficulties reaching a park, PBC becomes an important determinant of intention (14).

*H3:* Perceived behavioral control (PBC) is positively associated with behavioral intention (BI).

### 2.4 Behavioral intention (BI)

The behavioral intention measures the expressed intention, willingness, and effort toward the particular behavior (park visits) (15, 33). According to the TPB framework, intention is one of the most proximal predictors of behavior and serves as the motivational driver of the behavior (16). The analysis shows support for the link between intentions and behaviors; however, the predictive strength may depend on the way measurements are made and timed (34).

Studies employing TPB-based models have demonstrated that the intentions of the older adults are linked to park-use behavior in the TPB literature (14). Recently, a TPB-based study in the urban environment reported a significant influence of older adults park use behavioral intentions on park use behavior. Similarly, TPB models of park visit demand have demonstrated that intentions/demand predict park behavior, with attitudes, subjective norms, and perceived accessibility influencing them (17).

In Karachi, intentions will play an even more critical role since park visits require careful planning (for example, planning when and how to visit and whether to go alone or not). Therefore, intention plays an important role in mediating the effects of beliefs and constraints on park use behavior (17).

*H4:* Behavioral intention (BI) is positively associated with park-use behavior (BEH).

### 2.5 Park-use behavior (BEH)

Park-use behavior refers to realized visitation behavior, how often older adults visit parks, and how much time they spend there (35). It is analytically distinct from intention: someone may intend to visit but fail due to constraints, or may visit opportunistically without strong intention (36). Public-space and park scholarship increasingly emphasizes that social outcomes are not produced by physical space alone but by the ways spaces are used. A systematic review of public urban green spaces and social cohesion proposes that physical characteristics and perceptions may partly affect social cohesion through use patterns, suggesting that repeated presence and engagement can create conditions for social connection (19). Evidence among older adults indicates that factors such as accessibility, park features/conditions, and park use patterns relate to social interaction outcomes in parks (37).

Park-use behavior matters for social interaction because interaction opportunities require co-presence: being physically present in parks increases exposure to neighbors, acquaintances, and informal conversations, and longer durations increase the probability of interaction events (38). Studies examining social interaction mechanisms often operationalize interaction partly via frequency and duration of time spent with others in parks, reflecting this exposure logic (39).

In dense Karachi neighborhoods, parks may serve as one of the few non-commercial spaces where older adults can spend time outside the home; actual use frequency and duration are plausibly associated with whether social interactions occur in the park (40, 41).

*H5:* Park-use behavior (BEH) is positively associated with interaction in parks (SIP).

### 2.6 Social interaction in parks (SIP)

Social interaction in parks refers to interpersonal contact and engagement occurring in the park setting, such as talking with other visitors, meeting neighbors/friends, and perceiving that park visits increase one’s social interaction (11). Research increasingly distinguishes social interaction as a meaningful social-health pathway linked to public space (42). In systematic reviews on the relationships between public spaces and social cohesion, one learns that ordinary public spaces can enable social interactions through design aspects such as accessibility and furnishing, but depending on age and perceptions, such as perceived safety and comfort (9). Concerning older adults, there is specific evidence of how urban parks facilitate social interaction among older adults by establishing the importance of park attributes, situations, accessibility, and utilization (28). In fact, the conceptualization of parks as “spaces of encounter” makes an important contribution toward showing that parks are accessible spaces where older adults can make social contacts (18).

In Karachi, social interactions among older adults in parks are theoretically justifiable outcomes since they involve real and measurable social processes (social interaction), and parks have the potential to facilitate age-friendly goals such as enabling social connections and avoiding social isolation in places with mobility restrictions and weak civic infrastructure (11, 40, 43).

*H6:* Behavioral intention (BI) is positively associated with social interaction in parks (SIP).

### 2.7 Government and community support (GOV)

Community support and government action indicate older adults’ belief that government agencies and other community stakeholders contribute to maintaining parks, increasing their safety/usability, and providing efforts to increase their usability (25, 44). Government and maintenance of parks have been shown multiple times to be factors contributing to park usability. Visitor research emphasizes maintenance, safety perceptions, and the community atmosphere as factors that contribute to park access and usability (12). This suggests that the social environment and management conditions are elements that may affect access and usability (44). Regarding developing countries, urban green space governance has been defined as a complex issue, and participatory governance and institutional capacity have become factors influencing the accessibility and functionality of these spaces (45, 46). Pakistani studies on green infrastructure emphasize the role of communities and community-based indicators in sustainability in urban greenism (47).

In terms of TPB theory, these factors may be associated with individuals’ individuals’ beliefs regarding park control (“the parks are maintained,” “the parks are safe,” “the parks are usable”). As a result, these factors may by associated with perceived behavioral control of visitation (15, 22). It may be particularly relevant to Karachi, where there are significant differences in park maintenance and community support.

*H7:* Government and community support (GOV) is positively associated with perceived behavioral control (PBC).

### 2.8 Facilities and accessibility (FAC)

Facilities and accessibility capture perceived physical and functional conditions that enable older adults to use parks, including seating/rest areas, ease of access, and age-friendly facilities (24, 31). A substantial body of research shows that accessibility and amenities shape older adults’ experiences and outcomes in parks (8, 24, 31). Cross-level evidence suggests that park accessibility and amenities can moderate relationships between environmental perceptions, leisure activity, and wellbeing among older adults, indicating that usable facilities and access conditions shape what older adults can do in parks (7). Visitor studies also show that proximity, trails/paths, and maintenance concerns are prominent determinants of perceived access and desired improvements, reinforcing that facilities and accessible design are central to whether people experience parks as usable (23). TPB-based park-use models similarly highlight perceived accessibility as a determinant of demand/intention and behavior (17).

Within TPB, FAC is best positioned as an antecedent to PBC because it reflects perceived enabling conditions that directly inform “ease” and “control” beliefs about visiting parks (15, 17, 22). WHO age-friendly domains also emphasize outdoor spaces and buildings, accessible infrastructure, public seating, and sanitary facilities as part of supportive environments features that align closely with FAC as an enabling condition for older adults’ mobility and participation (1, 26).

*H8:* Facilities and accessibility (FAC) are positively associated with perceived behavioral control (PBC).

### 2.9 Mediation

The mediation hypothesis states that part of the influence of intention on social interaction occurs because intention increases actual park visitation, which is associated with greater exposure and opportunities for interaction. This mediation is conceptually consistent with TPB, which treats intention as the proximal antecedent of behavior (14, 15, 48). The second-step behavior leading to social interaction is supported by public-space and park literature, which argue that social cohesion and social interaction are shaped partly by use patterns (frequency, duration, and co-presence), and by older-adult park studies that find that park use patterns relate to social interaction outcomes (11, 19, 49). Therefore, a mediated pathway is theoretically coherent: intention is associated with visitation, and visitation is associated with interaction.

A partial mediation specification is also defensible. Intention may reflect planned social purposes (e.g., meeting someone in the park) and a proactive orientation toward engagement, which may be associated with interaction patterns beyond how often one visits (e.g., visiting at social times, choosing busier spaces, being open to conversation) (15, 36). This aligns with public-space evidence that social outcomes emerge from interactions among motivation, perception, and use rather than from use alone (9, 18, 19).

*H9:* Park-use behavior (BEH) statistically mediates the association between behavioral intention (BI) and social interaction in parks (SIP).

## 3 Research methods

### 3.1 Study design

This study employed a cross-sectional, questionnaire-based quantitative design to examine older adults’ urban park visitation and park-based social interaction in Karachi, Pakistan (50). The study was guided by a revised Theory of Planned Behavior framework in which attitude toward visiting urban parks, subjective norms, and perceived behavioral control were specified as predictors of behavioral intention (15, 16). Behavioral intention was specified as a predictor of park-use behavior and social interaction in parks, while government/community support and facilities/accessibility were specified as antecedents of perceived behavioral control (14, 15, 17). Park-use behavior was further tested as a mediator between behavioral intention and social interaction in parks (11, 51).

### 3.2 Study area

The study was conducted in Karachi, the capital city of Sindh Province, Pakistan. Karachi is Pakistan’s largest metropolitan center and one of the country’s most densely urbanized cities. According to the 2023 Population and Housing Census of Pakistan, Karachi Division had a population of approximately 20.38 million residents, reflecting substantial pressure on urban infrastructure, public services, and accessible public open spaces (52). The target population of this study comprised Karachi residents aged 50 years and older. Respondents were eligible to participate if they were aged 50 years or above, resided in Karachi, and were able to provide informed responses to the questionnaire. An age threshold of 50 years and older was selected instead of the conventional cutoff of 65 years frequently used in older adults studies from high-income countries, consistent with research conducted in low- and middle-income countries, where ageing-related health challenges often emerge at younger ages because of socioeconomic and environmental conditions (53). Furthermore, a recent systematic review reported that several studies examining the relationship between older adults, social interaction, and urban green spaces in developing countries also defined older adults as individuals aged 50 years and above (54). This definition is further supported by previous research suggesting that the lower age threshold of 50 years is more appropriate in developing-country settings, where exposure to lifelong hardship and limited healthcare may result in age-related health conditions appearing earlier than in more developed regions (55).

### 3.3 Sampling technique and data collection

The study used a citywide area-based purposive sampling strategy combined with door-to-door convenience recruitment (56, 57). Selected residential neighborhoods across Karachi were included to capture older adults’ experiences of urban park use in different parts of the city. The areas were purposively selected because they represented dense residential settings where neighborhood parks are relevant to everyday mobility, leisure, and social interaction among older adults.

Data were collected through face-to-face, door-to-door questionnaire administration. The field team visited households in selected residential areas and invited eligible residents aged 50 years and older to participate in the survey. This approach was suitable because the target population included older adults who may be less accessible through online surveys or public intercept methods (58, 59). After data screening, 576 valid responses were retained for analysis. A sample size of 576 was adequate for structural equation modeling. Prior literature generally regards a sample of around 300 as adequate for SEM, with 200 typically treated as the lower bound for stable parameter estimation using structural equation modeling (60, 61). The appropriate sample size is also known to depend on factors such as model complexity, the chosen estimation method, and the distributional properties of the observed indicators (62). A commonly applied rule of thumb sets the ratio of sample size to the number of free parameters between 5:1 and 20:1 (62–64). The measurement model in this study comprises 24 observed indicators across eight constructs. Applying a 10:1 ratio yields a minimum required sample size of 240. The final sample of 576 exceeds this threshold and is consistent with sample sizes recommended for models of comparable complexity (65).

### 3.4 Measures

The questionnaire operationalized eight constructs: attitude toward visiting urban parks, subjective norms, perceived behavioral control, behavioral intention, park-use behavior, social interaction in parks, government and community support, and facilities/accessibility. The Theory of Planned Behavior constructs were adapted from the original TPB framework and previous park-use behavior studies (14, 15, 17, 22). Attitude toward visiting urban parks was measured using three items assessing whether visiting urban parks is enjoyable, beneficial, and a good use of time (14, 15, 27). SN were measured using three items that captured perceived social approval and encouragement from important people, family members, close friends, and others around the respondent regarding regular park visitation (14, 15, 17). PBC was measured using three items assessing whether visiting urban parks is easy, whether respondents have enough time and ability to visit parks, and whether visiting parks is mostly under their control (14, 15). BI was measured using three items capturing respondents’ intention, planning, and effort to visit urban parks regularly (14, 15, 17).

Park-use behavior was measured using three indicators of actual visitation: frequency of park visits, average time spent in the park, and regularity of park visits (14, 17). Social interaction in parks was measured using three items assessing whether respondents often talk with other people in parks, whether parks provide opportunities to meet friends or neighbors, and whether visiting parks increases their social interaction with others (11, 18, 49). Government and community support was measured using three items assessing whether local authorities adequately maintain urban parks, whether community support improves park use and safety, and whether government and community efforts make parks more usable (23–25). Facilities and accessibility was measured using three items assessing whether urban parks have adequate seating and rest areas, whether parks are easy to access, and whether parks provide age-friendly and usable facilities (8, 23, 24).

### 3.5 Data analysis

The data were analyzed using structural equation modelling. Structural equation modelling (SEM) usually can be done using two approaches, partial least squares based structural equation modelling (PLS-SEM) and Covariance based structural equation modelling (CB-SEM) (66, 67). PLS-SEM was selected over CB-SEM for several reasons. First, PLS-SEM is recommended when the research objective is prediction and explanation of variance in key target constructs rather than confirmation of an overall theoretical model, which aligns with the exploratory, prediction-oriented nature of this study. Second, PLS-SEM does not require the observed variables to meet strict multivariate normality assumptions, unlike CB-SEM, making it more suitable for survey-based social science data of the type collected in this study (68). Third, PLS-SEM performs reliably with relatively complex models involving multiple constructs and structural paths, as in the present model (64, 69). Therefore, PLS-SEM is well suited to studies conducted in developing-country, where measurement instruments are often adapted from Western-developed scales and may not yet demonstrate the level of theoretical maturity assumed by CB-SEM. The measurement model was assessed using outer loadings, indicator VIF, Cronbach’s alpha, composite reliability, and average variance extracted (68, 70). The structural model was assessed using standardized path coefficients, t-values, *p*-values, bootstrapped bias-corrected confidence intervals, R-square, adjusted R-square, and f-square effect sizes, based on a bootstrapping procedure using 5,000 resamples (70–72). The indirect effect of behavioral intention on social interaction in parks through park-use behavior was tested using bootstrapping.

## 4 Results

### 4.1 Sample description

Table 1 presents the demographic and park-use characteristics of the 576 respondents. The largest age group was 50–55 years, representing 50.17% of the sample, followed by respondents aged 56–60 years at 33.68%. Smaller proportions were aged 61–65 years at 9.9% and above 66 years at 6.25%. The sample was male-dominated, with males accounting for 65.8% and females 34.2%, suggesting a possible gender imbalance in outdoor mobility or survey participation. In terms of education, nearly half of the respondents had secondary education at 49.13%, followed by primary education at 21.88%, no formal education at 15.45%, and graduation-level education at 13.54%. Household size was generally large, with the highest proportion living in households of five-members (33.33%), followed by four-member households (21.35%) and three-member households (18.58%).

**Table 1:** Demographic and park-use characteristics of respondents.

Demographics	Categories	Frequency (n)	Percentages (%)
Age	50–55	289	50.17
	56–60	194	33.68
	61–65	57	9.90
	≥66	36	6.25
Gender	Male	379	65.80
	Female	197	34.20
Education	Uneducated	89	15.45
	Primary	126	21.88
	Secondary	283	49.13
	Graduation	78	13.54
Household size	1	34	5.90
	2	59	10.24
	3	107	18.58
	4	123	21.35
	5	192	33.33
	6 or more	61	10.59
Frequency of visiting the park	0 day	91	15.80
	1–2 days	294	51.04
	3–4 days	132	22.92
	5–6 days	31	5.38
	7 days	28	4.86
Time spends	10–30 min	49	8.51
	31–60 min	106	18.40
	1 h − 2 h	125	21.70
	2 h-3 h	183	31.77
	3 h-4 h	76	13.19
	>4 h	37	6.42

*N* = 576.

Park-use patterns show that 51.04% of respondents visited parks one to two days per week, while 22.92% visited three to four days per week. A smaller proportion visited five to six days per week at 5.38%, and 4.86% reported daily visits. However, 15.8% reported no park visits. Regarding duration of park use, the most common stay was two to three hours at 31.77%, followed by one to two hours at 21.7% and 31–60 min at 18.4%.

### 4.2 Measurement model

The findings on the measurement model are provided in Table 2. It can be seen that all standardized factor loadings exceeded the established minimum of 0.70 (68). Thus, all loadings ranged from 0.834 (ATT1) to 0.951 (PBC1). High loadings indicate that all constructs are adequately measured by the selected indicators. Variance inflation factors ranged from 1.849 to 4.658, below the common threshold of 5.0 and indicating the absence of multicollinearity among the selected indicators (68). Furthermore, common method bias (CMB) was also assessed using Harman’s single-factor test, in which all indicators were entered into an unrotated exploratory factor analysis (73, 74). The results showed that the first factor accounted for only 25.40% of the total variance implying that common method bias is not a significant concern in this dataset. Internal consistency of all constructs was at the acceptable level as all Cronbach’s alpha were equal or exceeded the minimum required 0.70 (α = 0.823 to α = 0.932). The level of composite reliability was also acceptable, ranging from 0.907 to 0.956 (68). AVE ranged from 0.765 to 0.880, which was above the established minimum of 0.50 (75).

**Table 2:** Standardized factor loadings.

Construct	Item	Outer loading	Indicator VIF	Cronbach’s alpha	CR	AVE
ATT	ATT1	0.834	1.849	0.847	0.907	0.765
	ATT2	0.898	2.261			
	ATT3	0.891	2.138			
SN	SN1	0.920	2.879	0.885	0.929	0.813
	SN2	0.867	2.205			
	SN3	0.918	2.724			
PBC	PBC1	0.951	4.658	0.932	0.956	0.880
	PBC2	0.925	3.288			
	PBC3	0.938	4.073			
BI	BI1	0.902	2.547	0.882	0.927	0.809
	BI2	0.917	2.838			
	BI3	0.880	2.217			
BEH	BEH1	0.936	3.728	0.917	0.948	0.858
	BEH2	0.934	3.629			
	BEH3	0.909	2.810			
SIP	SIP1	0.901	2.477	0.864	0.917	0.786
	SIP2	0.875	2.128			
	SIP3	0.883	2.146			
GOV	GOV1	0.926	1.959	0.823	0.919	0.850
	GOV2	0.917	1.959			
	GOV3	0.913	1.939			
FAC	FAC1	0.930	3.181	0.895	0.935	0.827
	FAC2	0.911	2.898			
	FAC3	0.886	2.350			

#### 4.2.1 Discriminant validity

Table 3 shows discriminant validity results using both the Fornell-Larcker criterion (lower diagonal) and Heterotrait-Monotrait Ratio of Correlations (HTMT) values (upper diagonal). The square roots of AVE (bold, on the diagonal) ranged from 0.875 to 0.938, and in every case exceeded the inter-construct correlations values, which confirmed discriminant validity under the Fornell-Larcker criterion (75). The HTMT values in Table 3 are under the 0.85 threshold, meeting the acceptable threshold to achieve discriminant validity of constructs (68, 76).

**Table 3:** Fornell-Larcker criterion and HTMT assessment of discriminant validity.

Constructs	ATT	SN	PBC	BI	BEH	SIP	GOV	FAC
ATT	**0.875**	*0.201*	*0.024*	*0.520*	*0.186*	*0.286*	*0.037*	*0.033*
SN	−0.169	**0.902**	*0.030*	*0.470*	*0.175*	*0.318*	*0.030*	*0.033*
PBC	−0.01	−0.016	**0.938**	*0.323*	*0.063*	*0.138*	*0.606*	*0.474*
BI	0.453	0.419	0.293	**0.899**	*0.410*	*0.624*	*0.211*	*0.171*
BEH	0.165	0.158	0.058	0.369	**0.927**	*0.681*	*0.027*	*0.034*
SIP	0.248	0.28	0.123	0.545	0.606	**0.886**	*0.041*	*0.055*
GOV	0.01	−0.017	0.532	0.18	0.021	0.032	**0.922**	*0.329*
FAC	−0.019	0.025	0.434	0.152	0.031	0.05	0.282	**0.909**

Upper diagonal values represent HTMT; Lower diagonal values are correlation values; Diagonal and bold values represent square roots of AVE values.

### 4.3 Structural model results

All hypothesized direct associations were hypothesized to be positive and statistically significant. In terms of predicting behavioral intention, the standardized coefficient for attitude towards visiting urban parks is highest (*β* = 0.543), with subjective norms not far behind (*β* = 0.516); perceived behavioral control is another predictor of positive significance (*β* = 0.306). Behavioral intentions to use parks was positively associated with park-use behavior (*β* = 0.369). Behavioral intentions (*β* = 0.372) and park-use behavior (*β* = 0.469) were positively associated with social interaction in parks. Government/community support (*β* = 0.445) and facilities/accessibility (*β* = 0.308) were positively associated with perceived behavioral control (Table 4). It can be concluded that psychosocial motivation and contextual enabling conditions play an important role, but park use is the more proximate determinant of park-based social interaction (Table 4).

**Table 4:** Structural model results and hypothesis testing.

Hypothesis	Path	Beta	Boot SD	*t*-value	*p*-value	2.5% BC CI	97.5% BC CI	f-square	Decision
H1	ATT → BI	0.543	0.025	21.460	<0.001	0.493	0.591	0.640	Supported
H2	SN → BI	0.516	0.027	19.340	<0.001	0.462	0.565	0.576	Supported
H3	PBC → BI	0.306	0.025	12.240	<0.001	0.258	0.357	0.209	Supported
H4	BI→BEH	0.369	0.036	10.350	<0.001	0.293	0.435	0.157	Supported
H5	BEH → SIP	0.469	0.030	15.760	<0.001	0.410	0.526	0.371	Supported
H6	BI→SIP	0.372	0.032	11.450	<0.001	0.306	0.432	0.233	Supported
H7	GOV → PBC	0.445	0.031	14.170	<0.001	0.384	0.504	0.289	Supported
H8	FAC → PBC	0.308	0.033	9.450	<0.001	0.243	0.371	0.139	Supported

**Table 5:** Mediation result.

Effect	Path	Effect estimate	Boot SD	*t*-value	*p*-value	2.5% BC CI	97.5% BC CI	Interpretation
Indirect effect	BI - > BEH - > SIP	0.173	0.019	9.050	<0.001	0.136	0.212	Significant indirect effect
Direct effect	BI - > SIP	0.372	0.032	11.450	<0.001	0.306	0.432	Direct effect remains significant
Total effect	BI - > SIP	0.545	0.031	17.570	<0.001	0.481	0.600	Total effect significant

### 4.4 Mediation

This indirect association between behavioral intention and social interaction through park use behavior is positive and statistically significant [*β* = 0.173, 95% BC CI (0.136, 0.212)] (Table 5). The direct association between behavioral intention and social interaction remains significant even after controlling for the mediator (*β* = 0.372), and the total association was also significant (*β* = 0.545). This pattern is consistent with partial statistical mediation: park-use behavior accounts for a significant indirect association, while the direct association remains significant. The result should be interpreted as a model-based association rather than a temporally ordered causal mechanism.

## 5 Discussion

This study examined how the core components of the Theory of Planned Behavior (TPB), contextual enabling conditions, park-use behavior, and social interaction are associated among older adults in Karachi. Three findings are particularly important. First, attitude and subjective norms had the largest associations with behavioral intentions to use parks. Second, government/community support was more strongly associated with perceived behavioral control than facilities/accessibility. Third, park-use behavior had a larger association with social interaction than behavioral intention, while both the direct and behavior-mediated associations between intention and social interaction remained significant. Attitude had the largest standardized association with intention (*β* = 0.543), followed closely by subjective norms (*β* = 0.516), while perceived behavioral control also showed a meaningful association (*β* = 0.306). This pattern supports the central TPB proposition that behavioral intention is related to personal evaluations, perceived social expectations, and perceived control (15, 16). It is also consistent with previous TPB-based research showing the relevance of these constructs to older adults’ intentions to use urban parks (14). Although the coefficients were not formally compared through pairwise statistical tests, their numerical ordering suggests that favorable evaluations of park visitation and perceived social encouragement may be particularly relevant to visitation intentions in the Karachi sample.

The association between subjective norms and intention deserves particular attention. Healthy-aging and urban-green-space research emphasizes that maintaining social connections and participation in everyday community life is important for older adults (1, 5, 11). Previous studies similarly characterize parks as settings in which repeated encounters, informal conversations, and neighborhood relationships may occur (5, 18). Within Karachi, encouragement from family members, friends, neighbors, or peers may be especially relevant for older adults whose independent mobility is constrained. However, subjective norms should not be equated with social interaction itself. The present finding indicates that perceived social approval is associated with the intention to visit parks; it does not establish that such approval subsequently increases visitation or social participation.

The extended model also clarifies the contextual character of perceived behavioral control. TPB conceptualizes perceived control as reflecting both personal capability and the perceived controllability of the behavior (15). In this study, government/community support (*β* = 0.445) and facilities/accessibility (*β* = 0.308) were both associated with perceived behavioral control. Their inclusion therefore extends the conventional TPB model by locating perceived control within the physical and governance conditions surrounding park use. The larger coefficient for government/community support suggests that confidence in maintenance, safety, management, and community stewardship may be at least as relevant to perceived visitability as the availability of physical facilities. This is particularly important in a dense Global South city, where the presence of a nearby park does not necessarily mean that the park is perceived as safe, usable, or reliably maintained.

The association between intention and park-use behavior (*β* = 0.369) was significant but moderate, with intention accounting for only a limited proportion of variation in behavior. This result is consistent with, but does not directly demonstrate, the well-established intention–behavior gap in TPB research (34). TPB identifies intention as the most proximal motivational correlate of behavior, but intentions are more likely to correspond with behavior when individuals have adequate actual control and when intentions remain stable over time. For older adults in Karachi, actual park visitation may also be associated with unmeasured conditions such as extreme heat, illness, fatigue, caregiving responsibilities, transport availability, unsafe street crossings, or changing park conditions. These factors are theoretically plausible explanations rather than findings directly tested in this study. Moreover, because intention and behavior were measured at the same time, the study did not prospectively observe whether previously stated intentions were translated into subsequent park visits.

Park-use behavior had the largest association with social interaction (*β* = 0.469). This finding is consistent with literature suggesting that visit frequency, time spent in parks, and repeated co-presence are associated with opportunities for informal encounters and neighborhood social ties (19, 37). Among older adults, parks may provide accessible settings for meeting acquaintances, observing community life, and maintaining everyday social participation (48, 49). The result therefore connects the TPB intention–behavior sequence with healthy-aging literature by examining a social outcome beyond park visitation itself. The significant direct association between behavioral intention and social interaction (*β* = 0.372) requires more cautious interpretation. The behavioral-intention items measured plans to visit parks rather than an explicit intention to socialize. Therefore, the direct coefficient should not be interpreted as evidence that intention independently produces social interaction. It may reflect unmeasured social motivations, anticipated social benefits, previous positive encounters, or aspects of park use not fully captured by visit frequency, duration, and regularity. Reciprocal interpretation is also possible: older adults who already experience social interaction in parks may report stronger intentions to return. Concurrent self-reporting and shared-method variance may additionally contribute to the association. The findings are therefore compatible with two statistical routes, one involving park-use behavior and one remaining directly associated with intention, but do not establish two separate causal mechanisms.

The mediation findings reinforce this distinction. The indirect association through park-use behavior was significant (*β* = 0.173), while the direct association remained significant. This pattern is appropriately described as partial statistical mediation. It is consistent with the theoretical proposition that intention is associated with visitation and that visitation is associated with social interaction. However, causal mediation requires temporal precedence and stronger control of confounding than the present design provides. Future research should measure intention, subsequent park use, and later social interaction at separate time points, preferably supplemented by visit diaries, systematic observation, or mobility tracking. Overall, the findings indicate that understanding older adults’ park-based social interaction requires consideration of motivation, social expectations, perceived control, supportive urban conditions, and actual park use, while causal and temporal interpretations await longitudinal evidence.

Several limitations should be considered when interpreting these findings. First, the cross-sectional design precludes establishing temporal precedence and causal relationships; therefore, the direct, indirect, and mediated pathways should be interpreted as statistical associations rather than causal mechanisms. Second, the concurrent use of self-reported measures may have introduced recall bias, social-desirability bias, and common-method variance, while reported park use was not independently verified through observation or mobility tracking. Third, participants were recruited from selected neighborhoods in Karachi, and women and adults aged 66 years and above were underrepresented, limiting the generalizability of the findings to the wider older population. Finally, objective environmental conditions, including park quality, distance, thermal comfort, pedestrian accessibility, and neighborhood safety, were not measured. Future research should employ longitudinal or quasi-experimental designs, probability-based and demographically balanced samples, and objective environmental and behavioral measures to examine the proposed relationships more rigorously.

## 6 Theoretical contributions

This paper adds value to the existing body of knowledge in several ways. First, it builds on the theory of planned behavior by moving away from the traditional intention-to-behavior link toward downstream social outcomes, namely social interaction within parks. Thus, the paper suggests that older people’s engagement in urban parks is associated not only with visiting these facilities but also with opportunities for social interaction in urban areas.

In addition, this paper further refines the theory of planned behavior by considering the contextual nature of perceived behavioral control. In particular, this paper considers facilities and accessibility as well as governmental and community support as predictors of perceived behavioral control. This approach is justified because older individuals’ ability to use urban parks will be influenced by various factors, including their physical condition, social environment, and other aspects related to the provision of parks and urban areas.

## 7 Practical and policy implications

Urban parks should be treated as age-supportive infrastructure across Karachi, but implementation should begin in the neighborhoods included in this survey. The local government should complete a park-level audit of shade, seating, toilets, lighting, paths, entrances, crossings, security, and maintenance. Parks with service deficits and concentrations of residents aged ≥50 should receive priority. Given Karachi’s extreme heat and unsafe pedestrian conditions, upgrades should provide shaded rest points, level routes, toilets, drinking water, lighting, ramps, and traffic-calmed crossings at park entrances.

The local government should pilot this approach in the surveyed neighborhoods. Because government/community support was more strongly associated with perceived behavioral control (*β* = 0.445) than facilities/accessibility (*β* = 0.308), each pilot park should have a designated maintenance officer, monthly inspections, a complaint-response channel, and a stewardship group involving older residents. Physical upgrades should address Karachi’s heat and mobility constraints through shaded seating, level paths, ramps, toilets, drinking water, lighting, and safe crossings at park entrances. The strong associations of attitude (*β* = 0.543) and subjective norms (*β* = 0.516) with intention support morning/evening walking groups, peer-led activities, and family-supported visits. Because park-use behavior showed the strongest association with social interaction (*β* = 0.469), evaluation should track visit frequency, time spent in parks, perceived safety, facility usability, and opportunities for interaction. Monitoring should include women and adults aged ≥66, who were underrepresented, while the 15.8% reporting no park visits should serve as a baseline. Successful pilots could guide implementation across Karachi and other Pakistani cities.

## 8 Conclusion

The study tested an adaptation of the Theory of Planned Behavior to examine associations with adults’ park use and social interaction among 576 older adults aged 50 years or older in Karachi, survey data and partial least squares structural equation modeling. Attitude, subjective norms, and perceived behavioral control were positively associated with behavioral intentions to use parks. Both Park usage and intention were positively associated with social interaction. Government/community support and facilities/accessibility were positively associated with perceived behavioral control. A significant model-based indirect association between behavioral intention and social interaction was also observed through park use.

Overall, park use was associated with favorable perceptions, social support, perceived behavioral control, and supportive contextual conditions. Park use showed the strongest association with social interaction, while government/community support showed a stronger association with perceived behavioral control than facilities/accessibility. These findings support complementing facility-related strategies with broader social, governance, maintenance, and safety measures. Because the design is cross-sectional, the reported relationships should be interpreted as associations rather than causal effects.

## Data Availability

The raw data supporting the conclusions of this article will be made available by the authors, without undue reservation.
